# Sodium-glucose co-transporter 2 inhibitor therapy: mechanisms of action in heart failure

**DOI:** 10.1136/heartjnl-2020-318060

**Published:** 2021-02-26

**Authors:** Shruti S Joshi, Trisha Singh, David E Newby, Jagdeep Singh

**Affiliations:** 1 BHF Centre for Cardiovascular Science, The University of Edinburgh, Edinburgh, UK; 2 Department of Cardiology, NHS Lothian, Edinburgh, UK

**Keywords:** heart failure, heart failure with reduced ejection fraction, pharmacology, systemic review

## Abstract

Patients with type 2 diabetes mellitus are at a higher risk of developing heart failure compared with the healthy population. In recent landmark clinical trials, sodium-glucose co-transporter 2 (SGLT2) inhibitor therapies improve blood glucose control and also reduce cardiovascular events and heart failure hospitalisations in patients with type 2 diabetes. Intriguingly, such clinical benefits have also been seen in patients with heart failure in the absence of type 2 diabetes although the underlying mechanisms are not clearly understood. Potential pathways include improved glycaemic control, diuresis, weight reduction and reduction in blood pressure, but none fully explain the observed improvements in clinical outcomes. More recently, novel mechanisms have been proposed to explain these benefits that include improved cardiomyocyte calcium handling, enhanced myocardial energetics, induced autophagy and reduced epicardial fat. We provide an up-to-date review of cardiac-specific SGLT2 inhibitor–mediated mechanisms and highlight studies currently underway investigating some of the proposed mechanisms of action in cardiovascular health and disease.

## Introduction

Heart failure is a global pandemic and the current worldwide prevalence of heart failure is estimated at 64.34 million cases accounting for 9.91 million years lost due to disability.^1^ The prevalence of heart failure increases with age and is associated with other comorbidities like type 2 diabetes mellitus, hypertension and obesity. People with type 2 diabetes are 2.5 times more likely to develop cardiac failure than people without diabetes.^2^ Concerns regarding cardiovascular adverse events associated with type 2 diabetes have conventionally focused around atherosclerotic events, but with major improvements in the treatment of myocardial infarction, stroke and limb ischaemia over the past few decades, heart failure is increasingly being recognised as one of its earliest and most serious complications.^3^

The problems of heart failure associated with diabetes are compounded by the fact that several therapeutic glucose-lowering agents are either ineffective or harmful. Thiazolidinediones, such as pioglitazone, activate peroxisome proliferator-activated receptor gamma (PPAR gamma) to improve insulin sensitivity. However, they also cause increased sodium and fluid retention, thereby leading to a 42% increased risk of incident heart failure.^4^ In contrast, dipeptidyl peptidase 4 inhibitors appear to have a neutral effect on major adverse cardiovascular events although one agent of this class, saxagliptin, has been associated with a 27% increase in hospitalisation for heart failure and a 22% increase in cardiovascular mortality.^5^ A meta-analysis of more than 24 000 individuals from four large clinical trials reported that the use of insulin for type 2 diabetes was associated with a 27% increase in all-cause mortality and 23% increase in hospitalisation for heart failure.^6^ Looking at 100 000 individuals in the ‘real-world’ setting of an administrative database, such treatment was associated with an odds ratio of 2.02 for all-cause mortality and 1.42 for heart failure hospitalisation.^6^ Therefore, there is an unmet need for safer and more effective glucose-lowering therapies for patients with type 2 diabetes and heart failure.

### Sodium-glucose co-transporter 2 inhibitor therapy

Sodium-glucose co-transporter 2 (SGLT2) inhibitors are a new class of glucose-lowering drugs. They work by blocking the low-affinity, high-capacity SGLT2 protein located in the proximal convoluted tubule of the nephron. The SGLT2 protein is responsible for the resorption of approximately 90% of filtered glucose while the remainder is reabsorbed by SGLT1 proteins found on the distal part of the proximal convoluted tubule. SGLT2 inhibition results in glycosuria (and natriuresis as the protein is a co-transporter), thereby lowering plasma glucose concentrations.^7 8^ This mechanism is unique compared with all other glucose-lowering agents as it does not interfere with endogenous insulin or incretin pathways.

In recent cardiovascular outcome trials, SGLT2 inhibitors are associated with 30%–35% lower risk of hospitalisation for heart failure.^9–12^ Other glucose-lowering agents appear to be more potent than SGLT2 inhibitors, but fail to reduce cardiovascular risk, particularly with regard to heart failure outcomes. Moreover, although the glucose-lowering efficacy of SGLT2-inhibitor therapy declines at lower estimated glomerular filtration rates, its cardiovascular benefits are remarkably preserved, even in patients with renal impairment. This implies differing mechanisms of action in glycaemic control and cardiovascular risk reduction.^13^ These potential cardioprotective mechanisms remain incompletely understood and we aim to describe some of the key theories (table 1) in this review as this could pave the way for targeting future novel therapies for both heart failure and type 2 diabetes.

**Table 1:** Potential mechanisms of cardiovascular benefits associated with SGLT2-inhibitor therapy

Conventional mechanisms	Novel mechanisms
Diuresis and reduction in blood pressure	Improved myocardial energetics
Improved glycaemic control	Improved myocardial ionic homeostasis
Weight loss	Autophagy
Increase in red blood cell mass and haematocrit	Altered adipokine regulation

### SGLT2 inhibitor therapy and cardiovascular outcomes

In response to concerns about the increased cardiovascular risk associated with some treatments for diabetes, regulators in the USA and Europe mandated cardiovascular safety trials of all new hypoglycaemic drugs in 2008. In recent cardiovascular outcome trials, the glucagon-like peptide 1 receptor agonist (GLP-1 RA) and SGLT2 inhibitors have demonstrated reductions in cardiovascular events, reversing the trend of previous therapies. While the benefits with GLP-1RAs are associated with reduced atherothrombotic events like myocardial infarction and stroke,^14^ SGLT2-inhibitor therapies have demonstrated improved outcomes in heart failure^9 10 15^ (table 2). Interestingly, the reduction in heart failure hospitalisation was observed within months of randomisation, suggesting a different mechanism of effect from those of other glucose-lowering drugs which usually takes years to manifest.^9–12^ The consistent signal of improved heart failure outcomes in the early trials led to the ‘Dapagliflozin in Patients with Heart Failure and Reduced Ejection Fraction’ (DAPA-HF trial) which was specifically designed to evaluate the efficacy of the SGLT2-inhibitor therapy in patients with heart failure and a reduced ejection fraction, regardless of the presence or absence of diabetes. This trial showed that dapagliflozin reduces the risk of worsening heart failure or death from cardiovascular causes by 26%, regardless of diabetes status, suggesting that these benefits are independent of the drug’s glucose-lowering effect.^15^' More recently, results from the ‘Empagliflozin outcome trial in patients with chronic heart failure with reduced ejection fraction’ (EMPEROR-Reduced) trial showed that empagliflozin also reduced the composite risk of cardiovascular death and heart failure hospitalisation in patients with known heart failure with reduced ejection fraction regardless of diabetes status.^16^ In both of these trials, the risk reduction seen in their primary outcomes was principally driven by a reduction in hospitalisations for heart failure.^15 16^ Meta-analysis of these two trials shows consistency of effect of both dapagliflozin and empagliflozin with reductions in all the individual endpoints of all-cause death, cardiovascular death, hospitalisation for heart failure or decline in renal function.^17^ Thus, these striking benefits appear to be a class effect of SGLT2-inhibitor therapies. So, what is the mechanism of these marked benefits?

**Table 2:** Summary of large clinical trials of the SGLT2-inhibitor class

Study title	Study type	Number of patients enrolled	Follow-up duration (years)	Study population (key inclusion criteria)	Primary outcome	Key secondary outcomes
EMPA-REG OUTCOME^59^	RCT 1:1:1 Empagliflozin 10 mg vs empagliflozin 25 mg vs placebo	7028	3.1	Patients with: Type 2 diabetes mellitus Established CV disease Body mass index ≤45 kg/m^2^ Glomerular filtration rate (GFR) >30	Reduction in CV death, non-fatal myocardial infraction or stroke in empagliflozin group vs placebo (HR 0.86, 95% CI 0.74 to 0.99)	All-cause mortality lower in empagliflozin vs placebo: p<0.01 HF hospitalisation lower in empagliflozin group vs placebo (HR 0.65, 95% CI 0.50 to 0.85) HF hospitalisation and CV death (excluding fatal stroke) lower in empagliflozin group vs placebo (HR 0.66, 95% CI 0.55 to 0.79)
CANVAS trial^60^	RCT 1:1:1 Canagliflozin 300 mg vs canagliflozin 100 mg vs placebo	10 142	2.4	Patients with: Type 2 diabetes mellitus High CV risk	CV death, non-fatal myocardial infarction or stroke (HR 0.86, 95% CI 0.75 to 0.87)	Reduction in CV death and HF hospitalisation greater in patients with known heart failure (HR 0.78, 95% CI 0.67 to 0.91)
DECLARE-TIMI 58^61^	RCT Dapagliflozin 10 mg vs placebo	17 160	4.2	Patients with: Type 2 diabetes mellitus Established CV disease or multiple risk factors	CV death, non-fatal myocardial infarction or stroke (HR 0.93, 95% CI 0.84 to 1.03)	Reduction in CV death or HF hospitalisation in dapagliflozin vs placebo (HR 0.83, 95% CI 0.73 to 0.95) Reduction in HF hospitalisation (HR 0.73, 95% CI 0.61 to 0.88) Reduction in death due to renal or CV causes (HR 0.53, 95% CI 0.43 to 0.66)
VERTIS CV trial^62^	RCT 1:1:1 Ertugliflozin 5 mg vs ertugliflozin 15 mg vs placebo	8246	3.5	Patients with: Type 2 diabetes mellitus Established CV disease	CV death, non-fatal MI or stroke (HR 0.97, 95% CI 0.85 to 1.11)	Lower HF hospitalisation in ertugliflozin group vs placebo (HR 0.70, 95% CI 0.54 to 0.90)
DAPA-HF trial^63^	RCT Dapagliflozin 10 mg vs placebo	4744	1.5	Patients with: Symptomatic heart failure Left ventricular ejection fraction (LVEF) ≤40% 50% patients with type 2 diabetes	Reduction in CV death, urgent heart failure visit or HF hospitalisation in dapagliflozin group vs placebo (HR 0.74, 95% CI 0.65 to 0.85)	Fewer HF symptoms reported on KCCQ in dapagliflozin group vs placebo (HR 1.18, 95% CI 0.65 to 0.85) Reduction in CV death and HF hospitalisation (HR 0.75, 95% CI 0.65 to 0.85) Benefits seen in both diabetics and non-diabetics
EMPEROR-Reduced trial^64^	RCT 1:1 Empagliflozin 10 mg vs placebo	3730	1.5	Patients with: Chronic HF, NYHA class II/III/IV LVEF ≤40% HF hospitalisation within 12 months 50% patients with type 2 diabetes	CV death or heart failure hospitalisation, for empagliflozin vs placebo (HR 0.75, 95% CI 0.65 to 0.86)	Composite renal outcome (chronic haemodialysis, renal transplantation, profound sustained reduction in eGFR): reduced in empagliflozin group vs placebo (HR 0.50, 95% CI 0.32 to 0.77) All-cause mortality lower in empagliflozin group vs placebo (HR 0.92, 95% CI 0.77 to 1.10) Benefits seen in both diabetics and non-diabetics

CI, Confidence interval; CV, cardiovascular; HF, heart failure; HR, Hazard ratio; KCCQ, Kansas City Cardiomyopathy Questionnaire; MI, myocardial infarction; NYHA, New York Heart Association; RCT, randomised controlled trial.

### Conventional potential mechanisms of benefit with SGLT2 inhibition

The desirable cardiovascular effects seen with SGLT2 inhibitor treatment may be multifactorial, but key pathways involved are currently being explored. Some of the conventional theories are discussed in the following section.

#### Diuretic and antihypertensive effects

**Figure 1:**
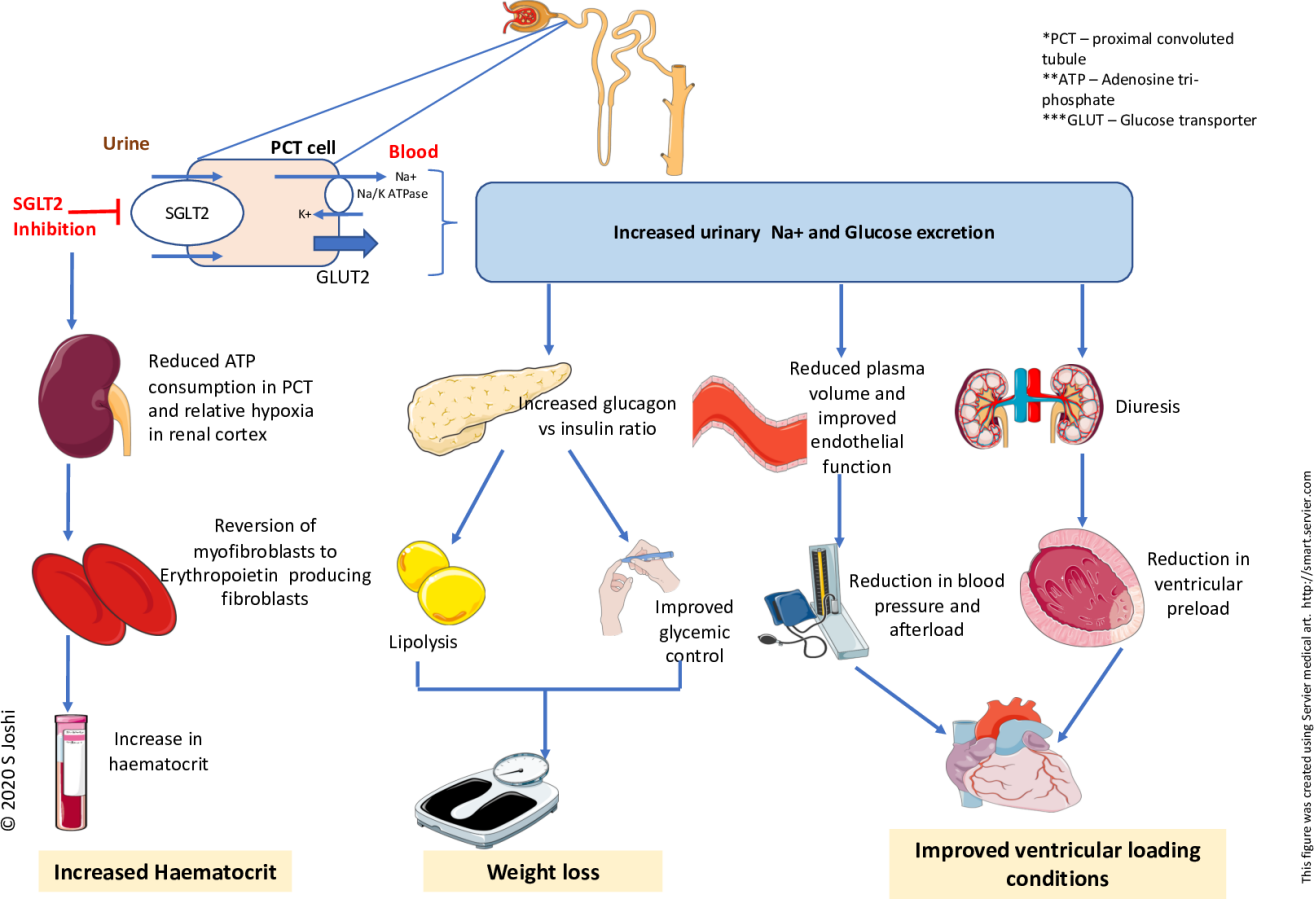
Schematic diagram showing conventional mechanisms of action of SGLT2 inhibitors. SGLT, sodium-glucose co-transporter.

It has been suggested that SGLT2 inhibitors improve cardiovascular outcomes due to their diuretic and antihypertensive effects (figure 1). SGLT2 inhibitors cause osmotic diuresis due to glucosuria and natriuresis, although the degree of diuresis and its composition (ie, the amount of natriuresis compared with aquaresis) remains to be established.^18^ Indeed, in the context of co-administration of loop diuretic therapies, the contribution of osmotic diuresis towards improved heart failure outcomes remains unclear. Glycosuria in the context of SGLT2-inhibitor therapy administration occurs due to a twofold to threefold increase in the filtered and resorbed glucose in presence of hyperglycaemia. Therefore, glycosuria and osmotic diuresis associated with SGLT2 inhibition are dependent on blood glucose concentrations and hence this does not explain the similar benefits observed in normoglycaemic patients with heart failure in the absence of diabetes mellitus.^15 16^ Although SGLT2-inhibitor therapy is associated with natriuresis and reduction in plasma volume,^9 19^ it is unclear if these benefits are sustained as there were no differences seen in serum N-terminal pro B-type natriuretic peptide (NT-pro BNP) concentrations in patients with chronic stable heart failure despite improvements in heart failure status.^20^ Moreover, there was no change in the diuretic dose among most participants during follow-up in the DAPA-HF trial, and the mean diuretic dose was similar between dapagliflozin and placebo groups.^15^

Recent studies have highlighted important differences between the diuretic effect of SGLT2 inhibitors and loop diuretic therapies. Using mathematical modelling, a recent study showed that both dapagliflozin and bumetanide were associated with a reduction in sodium and interstitial fluid. However, dapagliflozin had little or no effect on plasma volume whereas bumetanide was associated with reductions in intravascular volume, which can pose challenges in the context of hypoperfusion.^21^ This predicted selectivity of SGLT2 inhibitors for interstitial fluid is supported by observations from ‘the renal and cardiovascular effects of SGLT2 inhibition in combination with loop diuretics in patients with type 2 diabetes and chronic heart failure’ (RECEDE-CHF) trial which showed an average increase in daily urine volume of 545 mL, 312 mL of which was electrolyte-free water clearance.^22^ This may suggest a synergistic effect of SGLT2-inhibitor therapy with background diuretic treatment in heart failure. This, however, requires further validation.

The antihypertensive effect associated with SGLT2 inhibition was previously thought to be secondary to diuresis and natriuresis, but given that this effect is preserved even with declining glomerular filtration rates, it has been suggested to be more likely secondary to improved endothelial function, reduced arterial stiffness and changes in sympathetic nervous activity as evidenced by recent studies.^23 24^ A recent meta-analysis reported only a modest overall antihypertensive effect from SGLT2 inhibition: pooled estimate reduction of 2.46/1.46 mm Hg in blood pressure.^25^ This degree of reduction in blood pressure, although salutary in the context of cardiovascular disease, is also unlikely to be responsible for the striking benefits in cardiovascular morbidity and mortality.

#### Weight reduction and improved glycaemic control

It has been postulated that weight reduction and improved glycaemic control underlie the cardioprotective effect seen with SGLT2-inhibitor therapy. However, there are key considerations worthy of discussion. Weight loss from SGLT2-inhibitor therapy occurs due to an increased glucagon:insulin ratio causing increased lipid mobilisation and is thought to be one of the mechanisms involved in reduction in heart failure mortality associated with SGLT2-inhibitor therapy.^26 27^ Up to 2.7 kg weight loss was observed in patients with type 2 diabetes with SGLT2-inhibitor therapy, and there are some studies suggesting weight loss in pre-diabetic patients.^28 29^ However, there is currently no evidence to suggest that it can cause weight loss in patients with heart failure in the absence of diabetes which would be an argument against weight reduction being the primary mechanism of benefit with SGLT2-inhibitor therapy. Moreover, despite the high prevalence of obesity in heart failure, there is little definitive evidence regarding the impact of weight loss on cardiac function, quality of life and exercise tolerance in patients with heart failure.^30^ Therefore, weight loss alone cannot explain SGLT2 inhibition–related benefits in heart failure.

#### Increase in haematocrit

SGLT2-inhibitor therapy is associated with increases in renal erythropoietin production, red blood cell mass and haematocrit.^27^ Such changes may contribute to improvements in cardiovascular outcomes although similar increases in haematocrit have been reported with darbepoetin alfa and no mortality benefit was observed in patients with left ventricular systolic dysfunction.^31^

Overall, many of these mechanisms and recognised factors have been associated with reductions in cardiovascular risk (figure 1). However, the modest improvements seen with SGLT2-inhibitor therapy in these domains do not provide a clear explanation for the striking benefits observed in heart failure events in the large clinical trials and key pathways involved still require further investigation.

### Novel potential mechanisms of benefit with SGLT2 inhibition

#### Direct cardiac effects of SGLT2-inhibitor therapy

**Figure 2:**
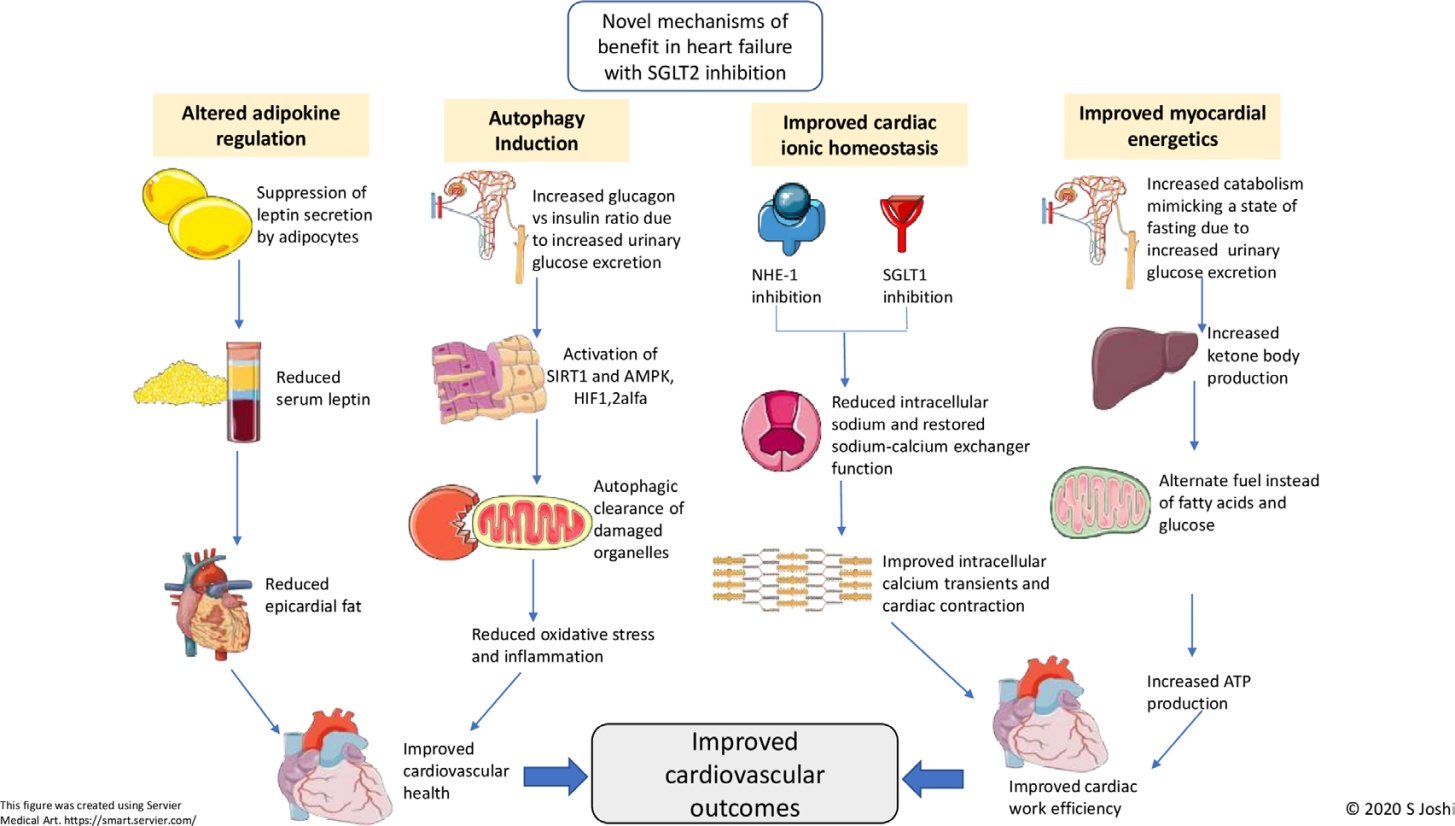
Schematic diagram showing proposed novel mechanisms of action of SGLT2 inhibitors in heart failure. AMPK, adenosine monophosphate-activated protein kinase; HIF, hypoxia-inducible factor; NHE, sodium-hydrogen exchanger; SGLT, sodium-glucose co-transporter; SIRT, sirtuin.

Cardiac hypertrophy, fibrosis and inflammation lead to adverse cardiac remodelling in heart failure, and this is a key contributor to its severity.^32^ Some pre-clinical and clinical studies have demonstrated a role for SGLT2-inhibitor therapies in reversing adverse cardiac remodelling.^33 34^ Although this effect has been observed in patients with type 2 diabetes and left ventricular hypertrophy, it has not been seen in patients with heart failure.^35 36^ This is intriguing, particularly, since the majority of its cardiovascular benefits have been around heart failure outcomes. This raises the possibility that, in the context of heart failure, SGLT2 inhibition may have novel direct cardioprotective effects, beyond that of ventricular loading and remodelling (figure 2).

### Improved myocardial energetics

Under physiological conditions, nearly 90% of cardiac energy is derived from mitochondrial oxidative metabolism and fuel is derived from free fatty acids, glucose and to a lesser extent from lactate, ketones and amino acids.^37^ In type 2 diabetes or heart failure, there is dysregulated fatty acid oxidation and impaired glucose uptake or oxidation causing myocardial dysfunction. In this setting of restricted fuel selection and low energetic reserve, ketone bodies are a ‘super-fuel’, producing ATP more efficiently than glucose or free fatty acids. SGLT2-inhibitor therapy increases hepatic synthesis and decreases the urinary excretion of ketones producing a mild and persistent state of hyperketonaemia. Under these conditions, beta-hydroxybutyrate (ketone body) is freely taken up by the heart and kidney and oxidised in preference to fatty acids and glucose. In mouse hearts, beta-hydroxybutyrate increases external cardiac work and reduces oxygen consumption, thereby improving cardiac efficiency.^38^ It has thus been hypothesised that the cardiovascular benefits of SGLT2-inhibitor therapy may be related to a shift in cardiac metabolism away from fatty acids and glucose oxidation towards more oxygen-efficient ketone bodies, thereby improving cardiac efficiency.

#### Improved ionic homeostasis in the myocardium

Calcium homeostasis in the myocardium is a finely balanced mechanism, essential for efficient excitation–contraction coupling.^39^ During systole, calcium is actively transported into the cardiomyocyte via L-type voltage-gated calcium channels where it binds to ryanodine receptors on the sarcoplasmic reticulum resulting in ‘calcium-induced calcium release’. This, in turn, activates calcium-sensitive contractile proteins (troponin C, troponin NC) which leads to myocardial contraction. In the setting of type 2 diabetes and heart failure, there is upregulation of both sodium-hydrogen exchanger 1 and SGLT1 resulting in markedly increased intra-cytosolic sodium content.^40 41^ This promotes calcium influx via the membrane bound sodium–calcium exchanger transporters and calcium efflux from the mitochondria (into the cytosol) via mitochondrial sodium–calcium exchanger transporters. The elevated baseline intracellular calcium content results in reduced calcium transients and smaller sarcoplasmic reticulum calcium stores in diabetic cardiomyocytes, thereby inhibiting contractile function.^42^

SGLT2-inhibitor therapy reduces cardiac cytosolic sodium content by inhibiting sodium-hydrogen exchanger 1 and SGLT1 transporters in diabetic rat and mice myocytes, thereby reversing calcium overload.^42–47^ Intriguingly, this effect on sodium-hydrogen exchanger 1 and SGLT1 is independent of diabetes status.^48^ These findings suggest that altered myocardial calcium handling is involved in the development of diabetic cardiomyopathy and heart failure, and that SGLT2-inhibitor therapy may improve the electrochemical characteristics in the failing myocardium which may contribute to its cardiovascular benefit. An ongoing clinical trial will assess the role of altered calcium handling in diabetic cardiomyopathy and heart failure and determine the effects of SGLT2-inhibitor therapy on cardiac calcium homeostasis (NCT04591639). The study uses a novel imaging approach called manganese-enhanced magnetic resonance imaging (MRI). Manganese acts as a calcium analogue and administration of the manganese-based contrast agent results in a marked shortening of the T1 relaxation times in myocardium with intact calcium handling. The rate of T1 shortening therefore acts as a measure of myocardial calcium handling, and this is the primary endpoint of the study.

#### Autophagy

Autophagy is the process by which cellular physiological equilibrium is maintained by removal of potentially dangerous constituents and recycling of cellular components as an adaptive response to metabolic stress including hypoxia and starvation.^49^ Experimental induction of autophagy has favourable effects in heart failure as it leads to efficient disposal of dysfunctional mitochondria, which are a major source of reactive oxygen species, promoting oxidative stress and inflammation.^50^ The pathways of autophagy induction involve activation of adenosine monophosphate-activated protein kinase (AMPK), sirtuin-1 (SIRT1) and hypoxia-inducible factors (HIF-1alfa and HIF-2alfa).^51^ It has been proposed that SGLT2 inhibitors may induce autophagy by simulating nutrient depletion by periods of increased catabolism due to constant glucosuria. Indeed, various SGLT2-inhibitor therapies have upregulated the expression of AMPK, SIRT1 and HIF-1 alfa.^52 53^ These actions might explain the phenomenon of autophagy and associated cardiovascular benefits with SGLT2-inhibitor therapy.^54^

#### Altered adipokine regulation

Leptin and adiponectin are cytokines produced exclusively by adipocytes. These ‘adipokines’ are essential in the regulation of food intake and energy homeostasis with leptin being implicated in various obesity-related cardiovascular diseases, whereas adiponectin is considered cardioprotective.^55^ Epicardial fat deposition due to altered adiponectin and leptin regulation is one of the theories implicated in the development of heart failure.^56^ Increased serum leptin concentrations are seen in patients with heart failure and they are associated with cardiac remodelling due to cardiac fibrosis and inflammation.^57^

SGLT2 inhibition reduces serum leptin and increases adiponectin concentrations, potentially offering some cardioprotection.^58^ However, these effects may well be reflective of changes secondary to the systemic effects of SGLT2-inhibitor therapy including weight loss and lipolysis.

### Future directions

The exact pathways of cardiovascular benefit with SGLT2-inhibitor therapy are yet to be established and newer mechanisms may emerge over time. It is plausible that SGLT2 inhibition interacts with or mediates other key pathways at a cellular level, facilitating cardiovascular benefits. Thus, establishing the exact mechanism of benefit is key to understanding the benefits of SGLT2 inhibition and may also open up new and unexplored pathways which could provide rich avenues of understanding for the pathophysiology and potential future novel treatments of heart failure.

There are several ongoing clinical studies investigating the mechanisms of cardiovascular benefits with SGLT2 inhibition such as the effects on cardiac remodelling (NCT03871621), lipolysis and modification of epicardial fat thickness and properties (NCT04219124, NCT04167761 and NCT02235298), myocardial calcium handling (NCT04591639) and endogenous ketone production (NCT03852901, NCT04219124).

These studies will provide significant insights into the key pathways involved in the cardioprotective effects associated with SGLT2-inhibitor therapy.

## Conclusions

SGLT2-inhibitor therapies are a promising new class of drugs for treating type 2 diabetes and heart failure. The cardioprotective effect of SGLT2 inhibition has been demonstrated in models of diabetic cardiomyopathy, heart failure and ischaemic cardiomyopathy. The mechanism through which SGLT2-inhibitor therapy exerts its benefit in patients with heart failure remains incompletely understood. It is possible that a combination of systemic and direct effects of SGLT2 inhibition on the myocardium ultimately leads to the cardiovascular benefits. However, there is strong pre-clinical evidence to suggest that improved myocardial ionic homeostasis has a role to play with regards to the cardiovascular benefits seen with SGLT2-inhibitor therapy. Studies examining the mechanistic role of SGLT2 inhibitor therapy in cardiovascular health will be instrumental in shaping our understanding of heart failure and diabetic cardiomyopathy and may open avenues for the development of future drug therapies that target these pathways.
