# Correction: *Sodium-glucose co-transporter 2 inhibitor therapy: mechanisms of action in heart failure*

**DOI:** 10.1136/heartjnl-2020-318060corr1

**Published:** 2021-10-26

Joshi SS, Singh T, Newby DE, *et al*. Sodium-glucose co-transporter 2 inhibitor therapy: mechanisms of action in heart failure. *Heart* 2021;107:1032–1038. doi:10.1136/heartjnl-2020-318060

This article has been corrected since it was first published. The provenance and peer review statement has been included.

